# Corticotropin-releasing hormone and extracellular mitochondria augment IgE-stimulated human mast-cell vascular endothelial growth factor release, which is inhibited by luteolin

**DOI:** 10.1186/1742-2094-9-85

**Published:** 2012-05-04

**Authors:** Shahrzad Asadi, Theoharis C Theoharides

**Affiliations:** 1 Molecular Immunopharmacology and Drug Discovery Laboratory, Department of Molecular Physiology and Pharmacology, Tufts University School of Medicine, 136 Harrison Avenue, Boston, MA, 02111, USA; 2 Department of Pharmacy, Tufts Medical Center, Boston, MA, 02111, USA; 3 Sackler School of Graduate Biomedical Sciences, Tufts University, Boston, MA, 02111, USA; 4 Department of Biochemistry, Tufts University School, Boston, MA, 02111, USA; 5 Department of Internal Medicine, Tufts University School of Medicine and Tufts Medical Center, Boston, MA, 02111, USA; 6 Department of Psychiatry, Tufts University School of Medicine and Tufts Medical Center, Boston, MA, 02111, USA

## Abstract

**Background:**

Autism spectrum disorders (ASDs) are neurodevelopmental disorders characterized by varying degrees of dysfunctional social abilities, learning deficits, and stereotypic behaviors. Many patients with ASDs have ‘allergy-like’ symptoms and respond disproportionally to stress. We have previously shown that the peptide neurotensin (NT) is increased in the serum of young children with autism and that can stimulate extracellular secretion of mitochondrial (mt)DNA which was also increased in the serum of these children.

**Methods:**

Human mast cells were stimulated by corticotropin-releasing hormone (CRH), mitochondrial DNA, IgE/anti-IgE, either for 24 hours to measure vascular endothelial growth factor (VEGF) release by ELISA or for 6 hours or quantitative PCR.

**Results:**

CRH augmented IgE/anti-IgE-induced human mast-cell release of VEGF and it also induced the expression of IgE receptor (Fc*ε*RI) on mast cells. Moreover, sonicated mitochondria also augmented VEGF release, and this effect was blocked by the natural flavone luteolin.

**Conclusion:**

These results indicate that stress and infection-mimicking extracellular mitochondrial components augment allergic inflammation that may be involved in the early pathogenesis of ASDs. Moreover, luteolin inhibits these processes and may be helpful in the treatment of ASDs.

## Introduction

Autism spectrum disorders (ASDs) are pervasive developmental disorders for which no distinct pathogenesis, biomarkers, or effective treatment have been identified. ASDs involve some immune dysfunction in the patient [1] or in the mother during gestation [2], and may have a neuroimmune component [3]. Many children with ASDs also have atopic features [4] or food allergies [5-7] that present as ‘allergy-like’ symptoms [7,8]. Such symptoms often occur in the absence of increased serum IgE levels or positive skin-prick tests, suggesting mast-cell activation by non-immune triggers [9]. Increased anxiety seems to be present in at least a subgroup of patients with ASDs, who may also be more prone to stress [10].

We previously showed that corticotropin-releasing hormone (CRH), secreted under stress, could induce release of vascular endothelial growth factor (VEGF) from human mast cells [11]. We found that the neuropeptide neurotensin (NT), which is present in both the brain and gut, is significantly increased in the serum of young children with autism [12]. It is interesting that the distribution of NT receptors is more concentrated in the brain Broca area [13], which regulates speech, a function commonly lost in children with autism [14]. We also found that the serum of the same patients had higher levels of extracellular mitochondrial (mt)DNA [15], and NT stimulated release of extracellular mtDNA from human cultured mast cells [15]. We also found that the natural flavonoid luteolin can inhibit the ability of IgE [16] and mercury [17] to induce VEGF release from human mast cells.

In the current study, we investigated the effect of CRH and mitochondria on VEGF release from IgE/anti-IgE-stimulated human mast cells, the effect of CRH on gene expression of the high affinity IgE receptor (Fc*ε*RI), and the effect of the flavone luteolin on VEGF release.

## Methods

The study was approved by the human institutional review board of Tufts Medical Center (Boston, MA, USA) under Exemption Number 4 for discarded samples without any identifiers.

### Culture of human mast cells

Human umbilical cord blood was collected from mothers who had normal uncomplicated deliveries at Tufts Medical Center. Human cord blood-derived cultured mast cells (hCBMCs) were prepared using hematopoetic stem cells (CD34^+^) isolated by positive selection of CD34^+^/AC133^+^ cells by magnetic cell sorting using an AC133^+^ cell isolation kit (Milletnyi Biotec, Auburn, CA, USA) as previously reported [18]. CD34^+^ cells were grown in serum-free expansion medium (StemSpan; StemCell Technologies, Vancouver, BC, Canada), supplemented with 100 ng/ml recombinant human stem cell factor (rhSCF; kindly supplied by Sweden Orphan Biovitrum AB, Stockholm, Sweden), 100 U/ml penicillin, 100 μg/ml streptomycin (Invitrogen, Carlsbad, CA, USA) and IL-3 (R&D Systems, Minneapolis, MN, USA) for the first 3 weeks, then in the serum-free expansion medium with 50 ng/ml IL-6 (Peprotech, Rocky Hill, NJ, USA) and for 8 to 10 weeks, with fetal bovine serum (Invitrogen/Gibco, Carlsbad, CA, USA) added from week 6. The purity of the hCBMCs was evaluated by immunocytochemical staining for tryptase [18]. hCBMCs cultured for 7 to 10 weeks were used for the experiments.

LAD2 cells (kindly supplied by Dr A.S. Kirshenbaum, National Institutes of Health, NIH, USA), derived from a human mast-cell leukemia cell line, were cultured in serum-free medium medium (StemPro®-34; Invitrogen) supplemented with 100 U/ml penicillin/streptomycin and 100 ng/ml rhSCF (Sweden Orphan Biovitrum AB, Sweden).

### Mitochondrial preparation

A commercial kit (Mitochondria Isolation Kit for Cells; Pierce Scientific, Rockford, IL, USA) was used to isolate mitochondria from cultured mast cells. Mitochondria were isolated under sterile conditions at 4°C in accordance with the manufacturer’s instructions, and then subjected to sonication for 2 minutes at 4°C to release all inner components. The mtDNA and protein concentrations were determined by UV spectrophometry (NanoDrop 2000; Thermo Scientific, Waltham, MA, USA). The purity of the mitochondrial fraction was confirmed by the absence of glyceraldehyde 3-phosphate dehydrogenase (GAPDH) and lactate dehydrogenase (markers of microsomal contamination) and of 5′ nucleotidase and glucose-6-phosphatase (markers of cytoplasmic contamination).

### Vascular endothelial growth factor release assay

VEGF secretion measured from LAD2 cells after pretreatment with CRH (10 μmol/l) for 24 hours, followed by 2 hours of incubation with IgE (1 microgram/μl) in response to anti-IgE (10 microgram/μl). Human mast cells were treated with IgE (1 μg/ml) for 2 hours (Millpore, MA, USA), then washed, and luteolin (100 μmol/l) was added for 30 minutes before stimulation with mitochondria (0.1 and 10 microgram/μl) and anti-IgE (10 μg/ml) (Dako, Carlsbad CA, USA). VEGF release was measured by ELISA (R&D systems, Minneapolis, MN, USA) in the supernatant taken from control and stimulated hCBMC cultures.

### Quantitative PCR

Total RNA from cultured mast cells and human skin biopsies was isolated using a commercial kit (RNeasy Mini Kit; Qiagen, Valencia, CA, USA) and reagent (Trizol; Invitrogen) respectively, in accordance with the manufacturer’s instructions. Reverse transcription was performed with 300 ng of total RNA using the iScript cDNA synthesis kit (BIO-RAD, Hercules, CA). To measure Fc*ε*RI expression, cells were incubated for 6 hours with CRH (Sigma-Aldrich, MA, USA) and quantitative PCR was performed using Taqman gene expression assays (Applied Biosystems, Foster City, CA, USA). Samples were run at 45 cycles using a real-time PCR system (7300; Applied Biosystems). Relative mRNA abundance was determined from standard curves run with each experiment. mRNA gene expressions were normalized to GAPDH endogenous control. (Hu, VIC TAMRA; Applied Biosystems)

### Statistical analysis

All experiments were performed in triplicate (n = 3) and repeated (n = 5). Results are presented as mean ± SD. Data from stimulated and control samples were compared using the unpaired two-tailed, Student’s t-test. p < 0.05 was considered significant.

## Results

**Figure 1:**
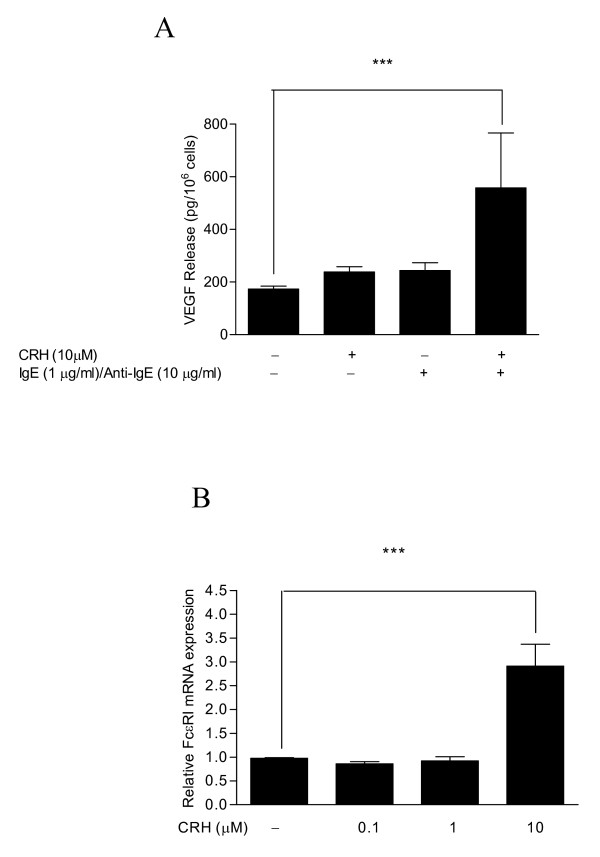
**CRH augments VEGF release from IgE/anti-IgE-stimulated human mast cells, and increases FcεRI gene expression**. (**A**) VEGF secretion from LAD2 cells was measured after pretreatment with CRH 10 μmol/l for 24 hours, followed by 2 hours of incubation with IgE 1 microgram/μl in response to anti-IgE 10 μg/ml. (**B**) FcεRI mRNA expression was assessed after stimulation of hCBMCs with CRH (0.1, 1, 10 μmol/l) for 6 hours. For all experiments, n = 5; *p < 0.05, **p < 0.01, ***p < 0.001 compared with control.

Because many children with autism have allergic symptoms are more anxious and over-react to stress, we investigated if CRH would affect allergic mast-cell activation. Addition of CRH (10 μmol/l) together with or after anti-IgE had no effect on anti-IgE-induced VEGF release (results not shown). Pretreatment of LAD2 cells for 24 hours with CRH (10 μmol/l) followed by 2 hours of incubation with IgE augmented VEGF release in response to anti-IgE (Figure 1A). The amount of CRH required was high because LAD2 cells do not express many CRH receptors.

We then investigated if such augmentation might be due to increase in Fc*ε*RI gene expression. Incubation of hCBMCs with CRH (0.1, 1, 10 μmol/l) for 6 hours increased Fc*ε*RI gene expression by almost five-fold (Figure 1B). Incubation of mast cells with CRH (10 μmol/l) for 48 hours increased Fc*ε*RI gene expression by almost 200-fold (results not shown).

**Figure 2:**
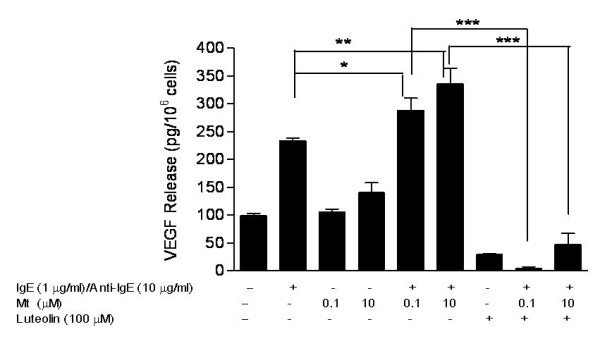
**Mitochondria augment VEGF release from IgE/anti-IgE-stimulated human mast cells, and inhibition by luteolin**. **(A)** VEGF secretion from hCBMCs was measured after pretreatment with IgE 1 microgram/μl for 2 hours and then incubating with mitochondria (0.1 and 10 microgram/μl) and anti-IgE (10 microgram/μl) for 24 hours. Pretreatment with luteolin 100 μmol/l for 30 minutes completely inhibited VEGF release and dropped it even below basal levels. For all experiments, n = 5; *p < 0.05, **p < 0.01, ***p < 0.001 compared with control.

We then investigated the effect of mitochondria. Treatment of hCBMCs with sonicated mitochondria (0.1 and 10 microgram/μl) stimulated some VEGF release (Figure 2), but addition of mitochondria to anti-IgE-stimulated mast cells significantly increased VEGF release (Figure 2). Pretreatment with luteolin (100 μmol/l) for 30 minutes completely inhibited the VEGF release induced by mitochondria and anti-IgE, and even caused it to drop below basal levels (Figure 2).

## Discussion

In this report, we show that CRH not only can augment allergic mast-cell release of VEGF, but can also induce Fc*ε*RI expression in these human mast cells. Our finding is specific, because the peptide substance P had been shown previously to decrease Fc*ε*RI gene expression [19], as also does lipopolysaccharide [20]. These results could explain how stress may worsen allergy-like symptoms in patients with ASDs [6,8,21]. It has previously been shown that CRH can augment NT-induced VEGF release [22]. Hence, CRH might augment both allergic and non-immune mast-cell activation. The mechanism of such augmentation was not known.

Increased anxiety seems to be present in at least a subgroup of patients with ASDs, who may also be more prone to stress [10]. A comparison of 34 adults with autism and 20 controls, matched for age, gender, and intellectual ability, found that patients with ASDs were three times as anxious as controls, and were significantly less able to cope with stress [23]. Acute stress can activate brain mast cells, an effect abolished by pretreatment with polyclonal antiserum to CRH [24]. Subsequently, CRH was reported to activate brain mast cells and increase blood–brain barrier permeability in rodents [25,26], particularly in brain areas containing mast cells [27]. The direct effect of CRH was documented by intradermal administration leading to increased vascular permeability in rodents and humans, through activation of CRHR-1 [28].

We also found that sonicated mitochondrial components at (10 microgram/μl) stimulates VEGF release, which also augments allergic stimulation of VEGF release from human mast cells. At the present, we are not sure which mitochondrial components are responsible for VEGF release. They may include ATP, mtDNA, or formyl peptides found in mitochondria. VEGF is also known to stimulate mitochondrial biogenesis [29], suggesting a possible paracrine effect on secreted VEGF on the mitochondria of neighboring cells.

Several studies have reported mitochondrial dysfunction in autism [30], which may involved a subset of children with autism [31,32]. Mitochondria are the primary energy-generating organelles in eukaryotic cells, and they participate in multiple intracellular processes, including calcium buffering [33]. However, mitochondria were originally bacteria that became symbiotic with eukaryotic cells, and are typically prevented from being released extracellularly by autophagy [34]. We previously found increased extracellular mtDNA in the serum of young children with autism [15]. The present results indicate that extracellular mitochondria components can augment allergic mast-cell stimulation. This action may be in addition to any direct effect that mitochondrial components may have on the immune system. For instance, damage-associated mitochondrial pattern are able to activate Toll-like receptor 9 on human peripheral polymorphonuclear leukocytes, leading to release of interleukin-8 [35].

Given that ASDs has been associated with brain inflammation and oxidative stress [1,3,36], we investigated the effect of the flavone luteolin, which has anti-inflammatory and anti-oxidant properties [37]. We found that luteolin 100 μmol/l was able to inhibit the augmenting effect at mitochondria on allergic human mast-cell activation. We used this concentration because it had been previously shown to cause maximal inhibition of mast cells and mast-cell-dependent stimulation of activated T cells [16]. Luteolin also blocks methyl mercury-induced VEGF release from human mast cells [17]. Myricetin, the structural analog of luteolin, can also inhibit mast-cell activation [38], and methyl mercury-induced mitochondrial dysfunction [39]. Luteolin also blocks activated peripheral blood mononuclear cells from patients with the inflammatory brain disease multiple sclerosis [40]. A new luteolin-containing dietary supplement was recently shown to have significant benefit in children with ASDs [41]. Luteolin may therefore be useful for the treatment of brain inflammation [40,42].

## Conclusion

Augmentation of allergic and mitochondria-stimulated mast-cell activation by CRH secreted by stress may explain at least some of the symptoms of patients with ASDs [43,44]. Other environmental triggers may also contribute ASDs [45,46], and to ‘mast-cell activation syndrome’ [47]. Luteolin may provide some degree of protection against these.

## Abbreviations

ASDs: Autism spectrum disorders; CRH: Corticotropin-releasing hormone; DPBS: Dulbecco’s phosphate-buffered saline; ELISA: Enzyme-linked immunosorbent assay; FBS: Fetal bovine serum; hCBMCs: Human umbilical cord blood-derived cultured mast cells; NT: Neurotensin; rhSCF: Recombinant human stem cell factor; VEGF: Vascular endothelial growth factor.

## Disclosures

TCT is the inventor of US patents Number 6,624,148; 6,689,748; 6,984,667, and EPO 1365777, which cover methods and compositions of mast-cell blockers, including flavonoids, US patents 7,906,153 and 12/861,152 (allowed) for treatment of neuro-inflammatory conditions, and US patent applications Number12/534,571 and Number13/009,282 for the diagnosis and treatment of ASDs. TCT is also the inventor of the dietary supplement, NeuroProtek®, which has the US trademark No 3,225,924.

## Competing interest

The authors declared that they have no competing interest.

## Authors’ contributions

TCT and SA prepared, read, and approved this manuscript.
